# Impaired sense of agency in functional movement disorders: An fMRI study

**DOI:** 10.1371/journal.pone.0172502

**Published:** 2017-04-27

**Authors:** Fatta B. Nahab, Prantik Kundu, Carine Maurer, Qian Shen, Mark Hallett

**Affiliations:** 1 Department of Neurosciences, University of California San Diego, La Jolla, CA, United States of America; 2 Departments of Radiology and Psychiatry, Mount Sinai Hospital, New York, NY, United States of America; 3 Human Motor Control Section, Medical Neurology Branch, National Institutes of Neurological Disorders and Stroke, National Institutes of Health, Bethesda, MD, United States of America; Centre National de la Recherche Scientifique, FRANCE

## Abstract

The sense of agency (SA) is an established framework that refers to our ability to exert and perceive control over our own actions. Having an intact SA provides the basis for the human perception of voluntariness, while impairments in SA are hypothesized to lead to the perception of movements being involuntary that may be seen many neurological or psychiatric disorders. Individuals with functional movement disorders (FMD) experience a lack of control over their movements, yet these movements appear voluntary by physiology. We used fMRI to explore whether alterations in SA in an FMD population could explain why these patients feel their movements are involuntary. We compared the FMD group to a control group that was previously collected using an ecologically valid, virtual-reality movement paradigm that could modulate SA. We found selective dysfunction of the SA neural network, whereby the dorsolateral prefrontal cortex and pre-supplementary motor area on the right did not respond differentially to the loss of movement control. These findings provide some of the strongest evidence to date for a physiological basis underlying these disabling disorders.

## Introduction

The sense of agency (SA) refers to our ability to exert and perceive control over our own actions [1]. SA has been extensively studied as a normal mechanism by which the brain monitors intended actions and their outcomes, regardless of the brain network being utilized [2–4]. Actions may include movements carried out by the motor system and thoughts. Regardless of the action, the SA network has been hypothesized to carry out this monitoring for a mismatch between the action that was intended and what has occurred [3,5,6] or will occur [7]. Those actions not matching their intended goal are detected passively and then relayed to consciousness where the individual is hypothesized to perceive a loss of SA or involuntariness of the movement or thought [4].

The neural correlates of the sense of agency have been explored using a variety of methods, including PET [6], fMRI [4,8–11], and EEG [12]. Despite the use of varying methods and paradigms, a common set of nodes are found across studies that include the pre-supplementary motor area (pre-SMA) [4,8–9,11,12], dorsolateral prefrontal area (DLPFC) [4,9,12], anterior insula [4,6,9,11], tempero-parietal junction (TPJ) [4,6,9,11,12], and precuneus/posterior cingulate [4,9,10]. While the role that each brain regions plays in SA remains poorly understand, there is experimental evidence that sheds light on the functions of each node. The roles played by the pre-SMA and SMA are hypothesized to include both motor intention and the experience of agency [8]. The right TPJ plays an important role in rejecting agency based on temporal sensory mismatch [6]. This role is further supported by work showing that parietal lesions were associated with impairment in the ability to evaluate internal vs. external derived visuo-motor feedback [13].

The vast majority of studies to date have focused on understanding SA in healthy individuals. Since SA is the basis by which control over ones actions is derived, its study is also relevant when characterizing individuals who lack this sense of control over their actions. We have previously hypothesized that there may be a clinical relevance to the perception of SA in individuals who experience actions originating from their own bodies and externally driven and have referred to such cases as ‘disorders of volition’ (DOV) [14]. Examples of DOV include auditory hallucinations in Schizophrenia (i.e. thoughts originating within the brain that are perceive as voices originating from the external environment), visual hallucinations in Parkinsonian disorders (i.e. seeing anything from a visual obscuration to a fully formed image of a person, etc. that has little to no basis based in the optical images coming to primary visual cortex from the eyes), passivity phenomena in Schizophrenia (i.e. a sensation that ones body is being moved by an external actor rather than the person’s own intentions), tics in Tourette syndrome (i.e. feelings of involuntariness that may be associated with the motor action of a tic movement), early chorea in Huntington disease (i.e. the presence of involuntary movements of the limbs that are involuntary that the affected individual initially assumes to be voluntary), and alien limb phenomenon whereby the affected limb will perform semi-purposeful or purposeful movements without the awareness or intention of the individual [14–15].

Another proposed example of a DOV is a poorly understood entity that is referred to in the medical literature by various names, including psychogenic movement disorder, motor conversion disorder, or our preferred term of functional movement disorder (FMD) that will be used hereafter. The clinical diagnosis of functional movement disorder (FMD) refers to the presence of subjectively reported involuntary movements that exhibit physiological characteristics of voluntary movement. The lack of understanding of FMD pathophysiology is further exacerbated by the paradoxical ubiquity and limited recognition of these disorders within neurological practices. Estimates range from 16% of new patients attending outpatient neurology clinics [16] to 20% of patients in movement disorder clinics [17] having a functional etiology.

Individuals with FMD can manifest a variety of movement types that often mimic neurological movement disorders such as tremor, parkinsonism, dystonia, tics, and myoclonus (see [18] for a review of this topic). The individual further reports that the movements are completely involuntary. On deeper inspection by a trained expert, these perceived involuntary movements demonstrate the characteristic features of voluntary movements, including variable or non-stereotyped movements, distractibility, entrainment (e.g. where movement characteristics such as tremor frequency or dystonic posturing cannot be maintained during contralateral and competing movements), or the presence of a Bereitschafts-potential by physiology [19]. These limitations on the motor system are only present during voluntary movements [20]. While FMDs may manifest similarly to other neurological movement disorders, they also lack the underlying physiology of the respective disorder (e.g. patients with functional parkinsonism have normal levels of brain dopamine).

This disparity between the individual’s report of having no control over these movements and the voluntary appearance of the movements has lead to the development a spectrum of awareness, using somewhat outdated psychiatric diagnostic classifications, ranging from conversion disorder (when the subject has a complete lack of awareness due to some psychic stressor or trauma) to malingering (when the subject is intending to deceive for personal secondary gain). The vast majority of FMDs are believed to be due to an underlying conversion disorder based on the use of expert-derived clinical criteria [21–22], though the diagnostic reliability of these criteria is poor [23] and no objective mechanism currently exists to differentiate perfectly. While the neural mechanisms underlying FMD remain poorly understood, some recent studies [24–25] have found an impaired sense of intention or agency within this population using behavioral paradigms. No studies to date have however looked at the neural correlates of SA in FMD, though a recent study comparing conversion tremor to voluntary or mimicked tremor did identify reduced activity of the right TPJ and suggested FMD may reflect abnormal integration of sensory prediction and feedback [26].

We previously developed an ecologically valid, virtual reality paradigm that could alter SA and allowed us to identify the involved brain networks using functional magnetic resonance imaging (fMRI) in a cohort of naïve healthy volunteers [4]. Those findings showed a broadly distributed and tightly integrated network of brain regions that were differentially activated based on the level of SA. In this paradigm, SA is implicitly judged while participants perform voluntary sequential finger movements and passively watched an artificial hand move with varying degrees of control. Based on those previous findings, we conducted an exploratory study of subjects fulfilling clinical criteria for FMD to determine whether their impaired sense of volition would demonstrate brain changes by fMRI in the SA network and on behavioral testing.

## Materials and methods

We utilized the same experimental paradigm, scan acquisition parameters, data analysis methods, and behavioral testing as previously described in for our healthy volunteer study [4]. A summary of the methods is provided below.

### Subjects

All aspects of this work were approved by the Institutional Review Board of the National Institute of Neurological Disorders and Stroke and all subjects provided written informed consent. All subjects were recruited from the Human Motor Control clinic within the National Institutes of Health in Bethesda, Maryland. Screening included a general medical evaluation, neurological testing by a trained movement disorder neurologist, and a clinical MRI to exclude latent structural lesions. Subjects were enrolled if they carried a diagnosis of clinically definite or clinically probable psychogenic movement disorder based on established criteria [21]. Subjects on psychoactive medications needed to be on a stable dose for at least 3 months prior to enrollment. To characterize the degree of involuntariness each subject experienced, they were asked to mark on a visual-analog scale ranging from fully involuntary (0) to fully involuntary (10) the degree of control they had over their predominant functional movement symptoms. Subjects were excluded if they were pregnant, had a contraindication for undergoing MRI or could not safely undergo MRI due to the severity of their movement disorder. Subjects were also instructed to abstain from caffeine and alcohol for 48 hours prior to scanning.

### fMRI procedures

We used a previously validated stimulus paradigm that allowed an adaptive quantitative modulation of SA. Briefly, all subjects wore a data glove on their right hand (regardless of their more affected side), which allowed measurement of individual joint positions for the entire hand, and they were asked to make sequential finger movements during the task period (30-seconds) that alternated with periods of rest (20-seconds). They observed a computer screen that displayed a moving hand that mimicked their movements completely (100% control), was completely random (0% control), or was an intermediate mixture (25%, 50% or 75% control). Data glove calibration and training were performed during complete mimic [4] for each subject with the goal of ensuring a sense of ownership over the projected hand and consistent performance across the groups. Two control conditions were also collected consisting of subjects watching a moving hand (W condition) and moving without visual feedback (M condition). Both conditions had been previously shown to activate primary brain regions (e.g. visual cortex and primary sensorimotor cortex, respectively) without generating significant activations of SA regions. Although we were studying individuals with FMD performing voluntary movements, subjects were told that they should return to making the voluntary finger movements even if a functional movement interrupted their voluntary movement during scanning. At the end of each scan, subjects were asked whether they had experienced any functional movements in the prior run. Similarly to our healthy volunteer cohort, we wanted FMD subjects to remain naïve to the goals of the study and the modulation of SA. They were informed that “the hand on the screen may not always do what you intend, but you should continue to perform your finger movements.” All movements were monitored online to ensure consistent performance across subjects/groups.

### Data analysis/statistics

Descriptive statistics were calculated for behavioral data. χ^2^ likelihood ratio testing was performed to test for group differences in the behavioral responses. Since the FMD group data collection lagged that of the healthy volunteer group, age-matching was not possible. To ensure that the findings were not confounded by age differences among the groups, we performed a Pearson’s correlation analysis between a metric of response error and age for all subjects (n = 41). The mean error was calculated as: Σ (subject reported control—actual control)/number of responses.

The fMRI data processing and analyses were carried out based on previously reported methods. Briefly, pre-processing included slice timing offset, 6-parameter rigid body motion correction, spatial blurring (6mm full width half-maximum Gaussian kernel) to minimize intersubject variability, and temporal normalization (dividing voxel signal intensity by the mean). A gamma-variate function [27] was used to model the hemodynamic response for each subject/task. For each voxel, the fixed shape analysis resulted in a single response amplitude for each stimulus class. We then performed a multiple linear regression analysis that included the regressors that modeled the stimulus response (0–100%) and regressors to model motion residuals and baseline drifts using quadratic polynomials in time for each run. The results of this analysis identified all brain regions that responded differentially to the modulation of SA. Statistical correction for multiple comparisons was set by rejecting spatial clusters smaller than would be expected by chance using Monte-Carlo simulations [28], given a voxel-wise false-positive level of *P* < 0.001 that resulted in a corrected *P* < 0.05 (minimum cluster size of 13 voxels, 351 μL). The individual subject maps were then used to generate a group map using a 2-way analysis of variance (ANOVA).

Since we were interested in comparing the network differences seen in the FMD group with those in our previously reported cohort, we performed a regions of interest (ROIs) analysis based on the areas found to be associated with our SA paradigm in the HV cohort [4]. The results therefore provide a comparison of the mean subject timecourse in each ROI for both the HV and FMD groups.

## Results

The demographics of the study groups are listed in Table 1. Fourteen of 21 subjects in the FMD group completed MRI data collection, with the remaining seven completing only behavioral testing due to unwillingness/inability to safely undergo MRI. Task performance was equal between the groups, with the FMD group showing full capability to carryout the sequential finger movement task during the course of the experiment. Only two subjects in the FMD group reported any functional movements during scanning, with the movements occurring ‘rarely’ by subject report and lasting a few seconds.

**Table 1:** Group demographics. Summary of age and gender of the FMD and HV groups.

	FMD Group	HV Group
Sample size (n)	21	20
Age ± SD	48.0 ± 11.0	23.6 ± 3.7
Gender (M:F)	9:12	10:10

Table 2 provides a summary of the clinical characteristics of the FMD group. The FMD group was comprised of a diverse sample of ages (range: 26–65), genders (9 males, 12 females), and symptoms. Features on examination that were consistent with the diagnosis of FMD included distractibility (76%), variable or non-stereotyped movements (67%), and motor entrainment with contralateral movement (67%). Depression symptoms were more commonly reported (76%) than anxiety (48%) or a history of abuse (19%). We also found additional comorbid disorders such as Fibromyalgia (19%), Bipolar disorder (documented mania) (14%), and post-traumatic stress disorder (PTSD) (9.5%).

**Table 2:** FMD group clinical characteristics. Gray areas represent findings on neurological examination that are consistent with FMD.

ID	Age	Symptom	SymptomaticSide/Region	Non-stereotyped	Distractable	Motor Entrainment	Depression	Anxiety	Abuse History	Other Diagnoses
P01	26	Tremor	Right hand	+	+	+				
P02	36	Myoclonus	Bilateral arms>body				+	+	+	
P03	63	Myoclonus	Right>Left legs	+	+	+	+	+		
P04	50	Ataxia	Bilateral (gait)				+	+	+	Bipolar II
P05	48	Tremor	Bilateral (arms/legs)	+	+		+			Fibromyalgia, Irritable Bowel Syndrome
P06	60	Tremor	Bilateral hands		+	+	+	+		
P07	64	Parkinsonism	Right hand			+	+	+		ADD
P08	45	Ataxia/ Myoclonus	Whole body	+	+		+			
P09	57	Tremor	Bilateral hands, head	+	+	+	+			Fibromyalgia, Chronic Fatigue Syndrome
P10	44	Tremor/ Myoclonus	Whole body	+	+	+		+		
P11	49	Myoclonus	Right>Left body	+	+		+			
P12	34	Dystonia	Left>Right body	+			+	+	+	Panic disorder, Eating Disorder, Bipolar II
P13	51	Tremor	Bilateral hands	+	+	+	+		+	PTSD, OCD, Bipolar, Codependent Personality Disorder
P14	50	Myoclonus	Right>Left body	+	+	+				
P15	29	Tremor	Bilateral arms	+	+	+				
P16	45	Tremor	Right>Left hand	+	+	+	+	+		
P17	57	Tremor	Right>Left body		+	+	+			
P18	49	Tremor	Right>Left limbs	+	+	+	+			
P19	49	Myoclonus	Left arm/leg		+	+	+			PTSD
P20	38	Tremor	Right arm	+	+	+		+		Fibromyalgia
P21	65	Myoclonus	Whole body	+			+	+		Fibromyalgia

### Behavioral results

**Fig 1:**
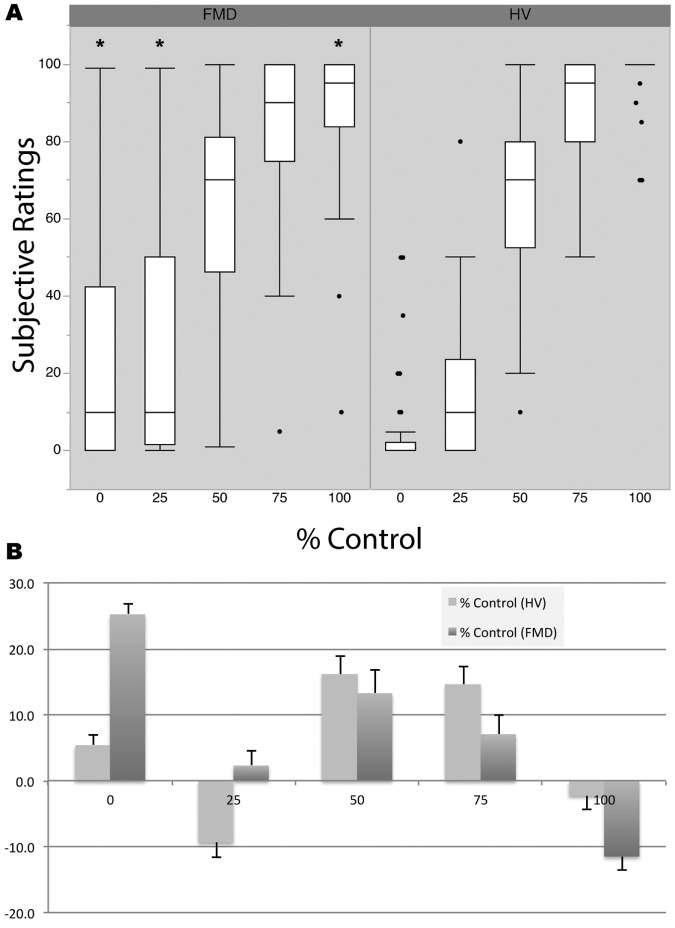
Behavioral data contrasting subjective SA with objective control among FMD subjects and controls. A) Box plots represent median subjective ratings (central line), upper/lower quartiles (box), min/max (whiskers), and outliers (points) for each group and control condition. B) Shows mean/standard error of group subjective ratings along with tendencies to over (positive) or underestimate (negative) control. Asterisks (*) represent significant differences (*p* < 0.05) between FMD and HV group responses.

Despite similar performance on the behavioral task at the completion of the fMRI data collection, the subjective ratings of % control between the two groups showed significant differences. Ratings within the FMD group were highly variable, particularly as control was lost (Fig 1A). While FMD subjects were better able to judge the 75% and 100% control conditions, they were still less accurate than controls. The FMD group showed similar bias tendencies to report higher levels of control during the 50% and 75% as our previously described HV group (Fig 1B). The FMD group differed from the HV as control was lost with a substantial proportion reporting continued SA over the data glove despite having lost most or all control under the 25% (χ^2^ = 27.31, *p* < 0.038) and 0% (χ^2^ = 28.05, *p* < 0.021) conditions, respectively. Particularly notable were the extremes where the patients claimed significant control when they had none and felt less than full control when control was complete.

Since our cohorts were collected in series with the FMD group lagging behind the HV group, we were unable to age-match. To ensure that age effects did not confound our observations, we calculated a Pearson’s correlation between a metric of error and age. The adjusted R^2^ was 0.009, providing strong evidence that the differences in behavioral data could not be explained by age effects.

### fMRI results

Whereas the behavioral task was designed to ensure paradigm validity by subjectively assessing SA experienced by participants at the end of fMRI data collection, the imaging data is intended to provide a direct assessment of how the brain responds to a loss of SA.

**Fig 2:**
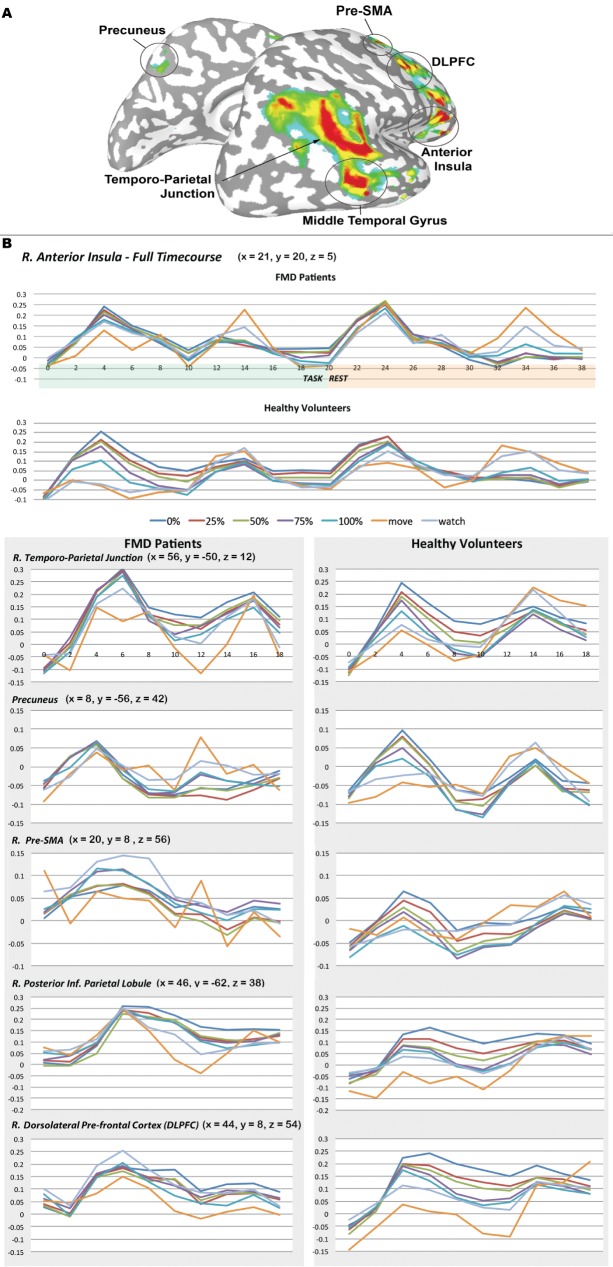
Comparison of fMRI responses to the modulation of self-agency in FMD group and controls. A) Linear trend map of regions responding proportionally to the loss of SA displayed on an inflated standard brain (*p* < 0.05, corrected). B) Mean BOLD response time courses [% signal change from the mean signal vs. time (sec)] for regions showing proportional responsivity to the level of control as SA was lost in FMD group and controls.

When compared to the HV cohort’s previous findings (Fig 2A), the FMD group showed similar spatiotemporal BOLD response changes that were modulated by the degree of control during a particular experimental condition (Fig 2B). We refer to a region’s differential and graded hemodynamic response changes based on the degree of SA as ‘stacking’. An example of this stacking can be seen by noting the magnitude of the BOLD response in the right TPJ of the HV group (Fig 2B). In this case, the magnitude of the BOLD response was: 0%>25%>50%>75%>100%>Watch>Move. Despite the similarities between the groups, several key differences were present. Generally speaking, the remarkable consistency of stacking in the HV group was present in the FMD group but was less clear and only in some regions, including the right anterior insula and right TPJ. Both of these brain regions notably have ‘leading’ response profiles, although the time to peak of the right TPJ showed a slight delay (6-sec) in the FMD group compared to HV group. Other ROI’s that did not exhibit the consistent stacking behavior including the precuneus (bilateral), right pre-SMA, right posterior inferior parietal lobule (IPL), and right DLPFC.

## Discussion

To evaluate whether individuals with FMD have impairments in their ability to judge their own intention or agency, we carried out an exploratory study to evaluate the regional blood flow responses collected by fMRI using a SA task previously carried out in a HV group to identify the brain regions modulated by changes in SA. Despite equivalent performance on the behavior task, the FMD group showed much greater variability in their perceived level of control and an unexpected tendency to overestimate control over the virtual hand. Furthermore as real-world control was lost, making the movements truly involuntary, the FMD group lacked the ability to recognize this loss. Although we originally hypothesized that individuals with FMD would consistently report having less control, our results showed the converse with the FMD group consistently over-reporting their %-control. These findings nonetheless provide additional weight to previous studies suggesting an impaired SA [24–25]. The validity of any subjectively reported metric is, however, questionable in a population known to inflate symptom severity and duration [29]. Although our two groups were not age-matched and the FMD group was older than the HV group (mean ages of 48.0 and 23.6, respectively), we found no correlation between age and the degree of error reported by subjects.

The neural network underlying SA is complex and widely distributed primarily over the right hemisphere of the brain. In our healthy volunteer cohort, we previously identified numerous brain regions that responded in a graded fashion to the loss of control during the implicit SA task. The findings within the healthy cohort are now strengthened by the results obtained from our FMD group.

Despite similar motor performance of voluntary movement and a lack of functional movements during the vast majority of the data collection, the hemodynamic responses of certain ROIs (right anterior insula and right TPJ) in the SA network of the FMD group demonstrated a reduced ability to be separated based on the %-control, while the remaining ROIs (right precuneus, right pre-SMA, right posterior inferior parietal lobule, and right DLPFC) showed no stacking whatsoever based on the degree of SA. Both the insula and TPJ ROIs were associated with the ‘leading’ response we identified in the HV cohort, while the remaining aberrant responses comprised ‘leading’ (right pre-SMA), ‘junction’ (precuneus), and ‘lagging’ (right IPL and right DLPFC) responses based on their temporal profiles and functional connectivities [4].

These findings are remarkable for at least two reasons. First, they suggest that although fMRI lacks the temporal resolution to provide information about directional connectivity, the identification of functionally intact regions with temporally earlier hemodynamic response profiles leading to downstream areas of dysfunction is indirect evidence of the direction of information flow and the possible site(s) of impairment. Second, the lack of hemodynamic stacking within our SA network in the FMD group strengthens the validity of our behavioral data since participants were naïve to the goals of the fMRI experiment and were only passively watching their finger movements rather than explicitly judging SA as during the behavioral experiment.

While it is beyond the scope of this manuscript to review the many and various functions attributed to these ROIs, these areas are known to be functionally connected based on human neuroimaging studies [30] and structurally connected based on non-human primate labeling studies [31]. Furthermore, these brain regions have been previously implicated in the ‘forward model’ framework for volition [15,32,33]. In this forward model, prefrontal and limbic areas are hypothesized to produce the drive to act. The motor program is generated by the pre-SMA, SMA and DLPFC, and this information is simultaneously relayed to primary motor areas and sensory integration regions (IPL and TPJ), via a corollary discharge, where intention and action are compared and volition is presumably determined.

While our findings lend support to the brain regions comprising the forward model of volition, questions arise about the direction of information flow that cannot be answered by a methodology like fMRI. For example, both the pre-SMA and DLPFC have a gradient of processing functions, whereby posterior sections manage motor actions and processing becomes more abstract and complex moving anteriorly [34]. Lau and colleagues [35] explored this connectivity while subjects performed a Libet judgment of the time of intended movement task [36]. They found that subjects attending to their intention to move exhibited higher levels activity and connectivity between the pre-SMA and DLPFC. Together the roles played by the pre-SMA and DLPFC have led some authors to propose that the “experience of intention requires proper connectivity” between these regions [37]. In this study the ‘W’ control condition demonstrated a different pattern between the groups in the region of the pre-SMA. In the HV group, the BOLD response pattern was near baseline as expected, while the BOLD response in the FMD group was higher than the other conditions for the latter half of the task. We speculate that this difference may represent the greater experience of SA in the FMD group that has been previously attributed to this region by others [8]. In the case of the forward model, both regions are associated with the selection and generation of the motor program, whereas in our study these areas show a differential responsivity to the degree of SA in the HV group that is no longer seen in the FMD group. This finding suggests that these regions play a larger role than the generation of the motor program and may be critical components for accurately judging volition. The DLPFC has previously been reported to be active when conflict between intention and sensory feedback arises [38]. The right IPL and precuneus have also been previously implicated in this network as areas responsible for mismatch detection [5].

The finding of fMRI-based differences in SA between individuals with FMD and controls expands upon previous behavioral and physiologic studies in this area. While these findings may not be surprising to some, there remains a large disconnect between the causes of FMD and the associated psychiatric features. In our own cohort, we found a substantial number of individuals (up to 76%) with a history of comorbid psychiatric disorders, with only a small minority receiving treatment with psychoactive medications. In many cases, the psychiatric symptoms had resolved while the FMD symptoms persisted. Many of our subjects also underwent detailed psychiatric assessments that found a higher frequency of early life stress and trauma, though failed to show proximate causation to the onset of functional symptoms and had similar rates of depression and anxiety to a control group with an ‘organic’ neurological disorder like focal hand dystonia [39].

Our findings of impaired hemodynamic responsiveness to changes in SA in critical areas of the network provide some of the strongest evidence to date for a physiological basis underlying FMD. While these findings need to be replicated and compared to an age-matched control group, future studies should focus on the roles played by different components of the network and how dysfunction in the network relates, if at all, to psychiatric comorbidities. Further work is also needed to replicate the stacking properties of the SA network, recognizing that substantially larger sample sizes will be needed to definitively show this. Our results also provide some of the first evidence for a trait-specific rather than state-specific etiology for FMD since participants were in large part not experiencing any functional movements during the course of the experiment. While we fully recognize the challenges of obtaining a homogeneous population using existed clinical criteria, this evidence does open the way for possible future genomics studies of FMD.

## Abbreviations

DOV: disorders of volition
FMD: functional movement disorders
fMRI: functional magnetic resonance imaging
